# Mental distress following inpatient substance use treatment, modified by substance use; comparing voluntary and compulsory admissions

**DOI:** 10.1186/s12913-016-1936-y

**Published:** 2017-01-03

**Authors:** Adrian R. Pasareanu, John-Kåre Vederhus, Anne Opsal, Øistein Kristensen, Thomas Clausen

**Affiliations:** 1 Addiction Unit, Sørlandet Hospital HF, Po. box 416, Kristiansand, Norway; 2 University of Agder, Kristiansand, Norway; 3 Norwegian Center for Addiction Research, University of Oslo, Oslo, Norway

**Keywords:** Compulsory admission, Substance use disorders, Mental distress, follow-up

## Abstract

**Background:**

Treatment services to patients with substance use disorders (SUDs), including those mandated to treatment, needs to be evaluated and evidence based. The Norwegian Municipal Health Care Act (NMHCA) calls for compulsory treatment for persons with “severe and life-threatening substance use disorder” if these individuals are not otherwise willing to be voluntarily treated and consequently risk their lives over drug use. Mental distress is known to be high among SUD patients admitted to inpatient treatment. The purpose of this study is to describe changes in mental distress from admission to a 6-month follow-up in patients with SUDs, which underwent either voluntary or compulsory treatment.

**Method:**

This prospective study followed 202 hospitalized patients with SUDs who were admitted voluntarily (VA; *n* = 137) or compulsorily (CA; *n* = 65). Levels of mental distress were assessed with SCL-90-R. Of 123 patients followed-up at 6 months, 97 (62 VA and 35 CA) had rated their mental distress at admission, discharge and follow-up. Sociodemographics and substance use severity were recorded with the use of The European Addiction Severity Index (EuropASI). We performed a regression analysis to examine factors associated with changes in psychiatric distress at the 6-month follow-up.

**Results:**

The VA group exhibited higher mental distress than the CA group at admission, but both groups improved significantly during treatment. At the 6-month follow-up, the VA group continued to show reduced distress, but the CA group showed increases in mental distress to the levels observed before treatment. The deterioration appeared to be associated with higher scores that reflected paranoid ideas, somatization, obsessive-compulsive symptoms, interpersonal sensitivity, and depression. Active substance use during follow-up was significantly associated with increased mental distress.

**Conclusion:**

In-patient treatment reduces mental distress for both CA and VA patients. The time after discharge seems critical especially for CA patients regarding active substance use and severe mental distress. A greater focus on continuing care initiatives to assist the CA patients after discharge is needed to maintain the reduction in mental distress during treatment. Continuing-care initiatives after discharge should be intensified to assist patients in maintaining the reduced mental distress achieved with treatment.

**Trial registration:**

ClinicalTrials.gov NCT 00970372 December 02, 2016.

## Background

Studies on the general population and clinical samples have consistently shown that psychiatric comorbidities are common among patients with substance use disorders (SUDs) [1–3]. Comorbidities have been associated with frequent psychiatric admissions [4], violence [5], suicidal behavior [6], poor treatment response [7, 8], poor long-term prognosis [9], severe impairments and disabilities [9], and high mortality rates [10], particularly among adolescents and young adults [9]. SUDs have also been positively correlated to different types of psychiatric disorders, such as depression or agoraphobia, and to the severity of the disorder [11]. Mental distress, defined as an individual’s level of mental complaints and symptoms, is frequently used as an outcome measure in medical and psychological research [12]. In screening for psychiatric disorders, the concept of mental distress is widely used. For example, it is estimated that among patients with SUDs between 30 and 50% suffer from a psychiatric disorder [13]. Other studies have shown that 30–40% of people with alcohol related disorders and 40-50% of people with other SUDs also have a psychiatric disorder [14–17]. In particular, patients with SUDs admitted to in-patient treatment showed even higher levels of mental distress [11, 18, 19] Overall, a SUD combined with a comorbid psychiatric disorder can have negative impacts on different aspects of patient conditions and functions [20].

Although SUDs are often difficult to cure, treatment methods are currently available to stabilize patients, reduce harm, and improve comorbidity. These effects can increase life expectancy and quality of life [21]. Traditionally, the goal of SUD treatment has been to achieve total abstinence, or at least reduced substance use. In general, reductions in substance use have been associated with improved outcomes of comorbid disorders [22]. For a treatment to be perceived as attractive and relevant to individuals with SUDs, it must provide worthwhile and rewarding experiences, in terms of reducing mental distress. Hence, treatments must benefit the patient’s perspective. To promote patient experiences of improvements, treatment services must be attentive to patient needs in the psychiatric domain, and monitor changes in clinically relevant outcomes throughout the course of treatment and following treatment [23]. There is consensus that patients with SUDs that have not responded to less intensive treatment efforts and whose SUDs’ poses an ongoing threat to their physical and mental health may require in-patient treatment [24]. Although in-patients are likely to experience reduced symptoms of mental distress, due to the controlled environment, little is known about the stability of symptoms over time, following discharge. Hence, positive improvements in mental distress that occur during treatment may not necessarily persist after discharge.

Voluntary admittance is the first choice and major gateway for treatment, but in the SUD field, voluntary admittance may not meet the expected positive outcomes, and patients may continue with detrimental patterns of substance use. In those circumstances, measures are available in many countries, including Norway, for applying compulsory in-patient drug treatment based on the medical needs of the patient, as opposed to resorting to legal means to coerce treatment through the criminal justice system.

The Norwegian Municipal Health Care Act (NMHCA) sanctions involuntary interventions for adult patients with SUDs in Norway [25]. The Act covers an option for retention (up to 3 months), when the health of the patient is seriously at risk, due to extensive, prolonged substance use, and when voluntary efforts have proven insufficient.

Literature reviews regarding compulsory treatment have generally concluded that research on the efficacy is inconsistent and inconclusive [26–29]. This is in part because these literature reviews do not always distinguish between different forms of compulsory treatment in conducting their analyses of outcomes. Most of the research and evidence on the effectiveness of compulsory treatment relates to offenders who are coerced and referred via the criminal justice system [30]. Among countries with a distribution of welfare through the state and in favor of using the civil law, Sweden has provided some relevant research in the field of patients with SUDs compulsory admitted to treatment. For example, Gerdner and Berglund concluded that patients compulsory admitted to SUD treatment have higher retention rates in treatment programs and aftercare, compared to VA patients. CA patients showed global outcomes that were as good as, or even better than those of VA patients[31]. Generally, for patients with SUDs and co-occurring psychiatric disorders admitted to treatment, it is particularly important to examine the outcomes at some point after the initial treatment episode has ended given that treatment effects may not have been retained during the follow-up phase [32]. There is a scarcity of follow-up studies that include both measures of substance abuse and mental distress for patients with SUDs [33]. Among patients with SUDs that underwent compulsory admission (CA), most studies examined treatment completion, reductions in substance use and less frequently, improvements in psychological symptoms [34–36]. Thus, a knowledge gap exists.

This study aims to: (1) describe the level of mental distress among a cohort of SUD patients; (2) examine the change in mental distress during the observation period by voluntary and compulsory treatment status; (3) analyze factors associated with change in mental distress at 6-month follow-up.

## Methods

### Settings and procedures

The Norwegian Social Services Act of 1993 allowed compulsory admissions to the hospital for persons with severe and life-threatening substance use. In 2012, this law was replaced by the NMHCA, §10.2, sanctions involuntary interventions for adult patients with SUDs [25]. In this manuscript, we followed a similar methodology to one used previously [37]. Recruitment for this prospective study continued consecutively between January 2009 and May 2011 from three different publicly funded treatment centres in the south-eastern part of Norway. The treatment wards had multidisciplinary staffs, including psychiatrists, psychologists, social workers, occupational therapists, specialized nurses, and other trained staff. The centres offered treatment for patients with a primary SUD, often combined with mental disorders. Treatment included assessment of somatic and mental health along with pharmacotherapy, cognitive milieu therapy, and individual motivation enhancement. Before study inclusion, the patients were detoxified, verified by negative urine tests for alcohol, opioids, central stimulants (amphetamines, methamphetamines, and cocaine), benzodiazepines, and cannabis to establish baseline values not influenced by withdrawal symptoms.

No formalized aftercare service was provided by the wards, but aftercare plans for individuals were made in collaboration with primary care services in the local municipalities; e.g., appointments with social services. Follow-up interviews were performed 6 months after discharge from the hospitals and took place between July 2009 and December 2011. Because patients came from all over the country (particularly the CA group), the project staff attempted to contact all patients directly by phone, mail, or post. In some cases, patients were found to be in prison or in inpatient treatment institutions and arrangements were made to meet them there, which included extensive travelling for the data collection team as all the interviews were conducted face to face.

### Participants

A total of 326 patients consecutively admitted to substance use disorder and psychiatry wards were identified as potentially relevant for this study. Participants were eligible when they were >18 years of age, had a SUD, could understand Norwegian, and were admitted at least 3 weeks prior to study inclusion, which allowed them sufficient time for detoxification and stabilization before providing informed consent. We verified whether the patients were detoxified, by negative urine tests for alcohol, opioids, central stimulants (amphetamines, methamphetamines, and cocaine), benzodiazepines, and cannabis; thus, we were able to establish baseline values that were not influenced by withdrawal symptoms. According to the inclusion criteria, 228 were eligible, but 26 patients refused to participate. Of the 202 patients enrolled at baseline (65 CA and 137 VA), 123 (61%) were followed-up at 6 months.

Because of limitations in funding and the large geographical uptake area, CA patients were prioritized for follow-up (82% CA patients versus 59% VA patients were included) because the CA patients were less represented in the sample at baseline. Thus, the higher loss to follow-up in the VA group was mainly due to administrative and logistic reasons. Beyond this, the attrition analysis showed no other differences between those who were and were not included at follow-up in terms of demographic data, severity scores, or length of stay in the initial treatment episode.

### Instruments and measure

The Mini International Neuropsychiatric Interview, version 5.0, was conducted at baseline to assess SUD and other psychiatric diagnosis [38]. For statistical purposes, psychiatric diagnoses were categorized as Axis I (symptom disorders) and Axis II (personality disorders). Common Axis I disorders include anxiety and mood disorders, attention deficit disorders, schizophrenia and other psychotic disorders.

To assess demographics and severity of substance use variables, the most commonly used measure within addiction treatment research was used: The European Addiction Severity Index (EuropASI) [39, 40]. The EuropASI is a structured interview performed by trained and certified staff. The same questionnaire was used at follow-up. Drug and alcohol use in the 30 days preceding the follow-up interview were evaluated to determine whether the patients were abstinent or not. Additionally, time in a controlled environment as defined by the EuropASI as days in jail or SUD treatment in the 30 days before follow-up was used to assess differences in engagement with aftercare services. As a proxy for severe mental distress, we assessed whether patients had ever had suicidal attempts in their lifetime. Mental distress in general was measured with the Symptom Checklist-90-R (SCL-90-R) [41], a widely used inventory used clinically in Scandinavia to monitor psychological distress both before and after treatment [42–44]. Additionally, the SCL-90-R has been tested in a Norwegian population sample [45]. The SCL-90-R has 90 items rated on a five-point Likert-type scale, ranging from “not at all” (0) to “extremely” (4), and includes nine subscales (somatization, obsessive-compulsive, depression, anxiety, hostility, interpersonal sensitivity, phobic anxiety, paranoid ideation, and psychoticism). The present study uses the Global Severity Index (GSI), which is the average rating of all 90 items. GSI is often used as an overall index of distress in studies of substance dependent samples [46]; the higher the score, the greater the distress [47]. A score of GSI > 1 is considered to be a pathological score. Changes in mental distress were computed by subtracting the GSI determined at follow-up from the GSI determined at admission, hereafter called the ‘GSI change’. Thus, a ‘positive score change’ refers to an improved mental distress. For this study, we chose to include only the participants that provided complete dataset on the outcome variable, mental distress.

### Analysis and statistical methods

Descriptive statistics were used to elaborate baseline characteristics. To examine the change in psychiatric distress between discharge and the 6-month follow-up, we used the paired sample *t*-test, for both CA and VA groups. Linear regression was used to examine predictors of changes in psychiatric distress, from baseline to the 6-month follow-up. Preliminary bivariate analyses were first undertaken. Variables with seemingly little influence on the dependent variable (p-value >0.20) were not included in the multiple regression (adjusted model), as recommended by Altman [48]. We also controlled for gender and age. The final multiple regression model used simultaneous entry of variables (the “enter” method). Results are presented as unstandardized beta coefficients with 95% confidence intervals (CIs). P-values < 0.05 were considered statistically significant. Analyses were performed with SPSS 21.0 Software (SPSS Inc., Chicago, IL, USA).

## Results

**Fig. 1:**
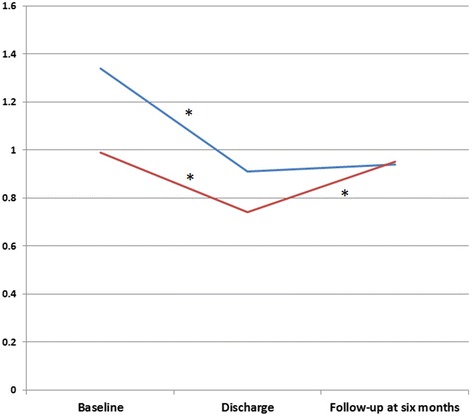
Changes in mental distress from baseline to 6-month follow-up in patients treated for substance use disorders. Notes: T1 = baseline, T2 = discharge, T3 = follow-up at 6 months. Red line: changes in mental distress in voluntary admission group. Blue line: changes in mental distress in compulsory admission group. Mental distress was measured with the Global Score Index (GSI) of the Symptoms Checklist (SCL-90-R). * *P* value <0.01

At baseline, the 202 participants had a mean age of 30 years, and 34% were women (Table 1). Among these, 32% (*N* = 65) had undergone CA and 68% (*N* = 137) were VA. All patients met the ICD-10 criteria for SUDs; the majority had a drug use disorder (83%). Use of an injected drug 6 months prior to hospitalization was reported by 54% of participants. The mean SCL-90 GSI for our cohort, at baseline, was 1.2, which is 0.2 above the pathological cut off used as a general measure of psychopathology at the group level. In our cohort, 56% of patients had scores above the pathological cut-off. The burden of mental distress was higher in the VA group than in the CA group, mean difference (MD) = 0.34, t(95) = 2.34, *p* < 0.02, CI: 0.053-0.632, but distress improved at similar magnitudes between groups during treatment (Fig. 1). At the end of treatment, the majority of patients (67%) had reduced their mental distress scores to below the clinical cut-off for pathology. Of the 123 patients followed-up at 6-months, 97 (35 in the CA group and 62 in the VA group) had rated their mental distress at all three time points (at admission, discharge, and the 6-month follow-up); these were included in the follow-up analyses. An attrition analysis showed no other differences between those who completed the SCL-90-R interview and those who did not, in terms of demographic data, severity scores, or levels of mental distress.

**Table 1:** Baseline socio-demographic variables for patients with SUDs

Variables	All patients, *N* = 202	Follow-up sample, *N* = 123
Mean age, years	30.0 (±8.9)	30.4 (±9.8)
Female	68 (34)	47 (39)
Education, years	10.8 (±1.9)	10.8 (±2.1)^a^
Relationship status, single	136 (69) ^b^	84 (69)^c^
Main SUD diagnosis		
Alcohol use disorder (AUD) or AUD + concurrent drug use disorders	34 (17)	20 (16)
Drug use disorder	168 (83)	103 (84)
Psychiatric diagnosis		
Axis I disorders	117 (60)	71 (58)
Axis II disorders	9 (4.5)	4 (3)
Axis I and II disorders	24 (12)	16 (13)
Only SUD diagnosis	52 (26)	32 (26)
Severity scores		
Injection use	105 (54) ^d^	71 (60)^e^
Duration of most problematic substance use, years	11.1 (±7.6)	11.6 (±7.6)^f^
Time in treatment, days	57 (26)	58 (26)
Suicidal attempts – lifetime prevalence	94 (49) ^g^	60 (51)^h^
Compulsory admission	65 (32)	51 (42)
SCL-90-R GSI, mean (SD)	1.2 (±0.70)^i^	1.2 (±0.74)^j^

Values represent the numbers of patients (%) or mean (±SD); in some cases, the total number of patients changed, as follows: ^a^ *N* = 117; ^b^ *N* = 198; ^c^ *N* = 121; ^d^ *N* = 195; ^e^ *N* = 119; ^f^ *N* = 117; ^g^ *N* = 191; ^h^ *N* = 117; ^i^ *N* = 197, ^j^ *N* = 120

**Fig. 2:**
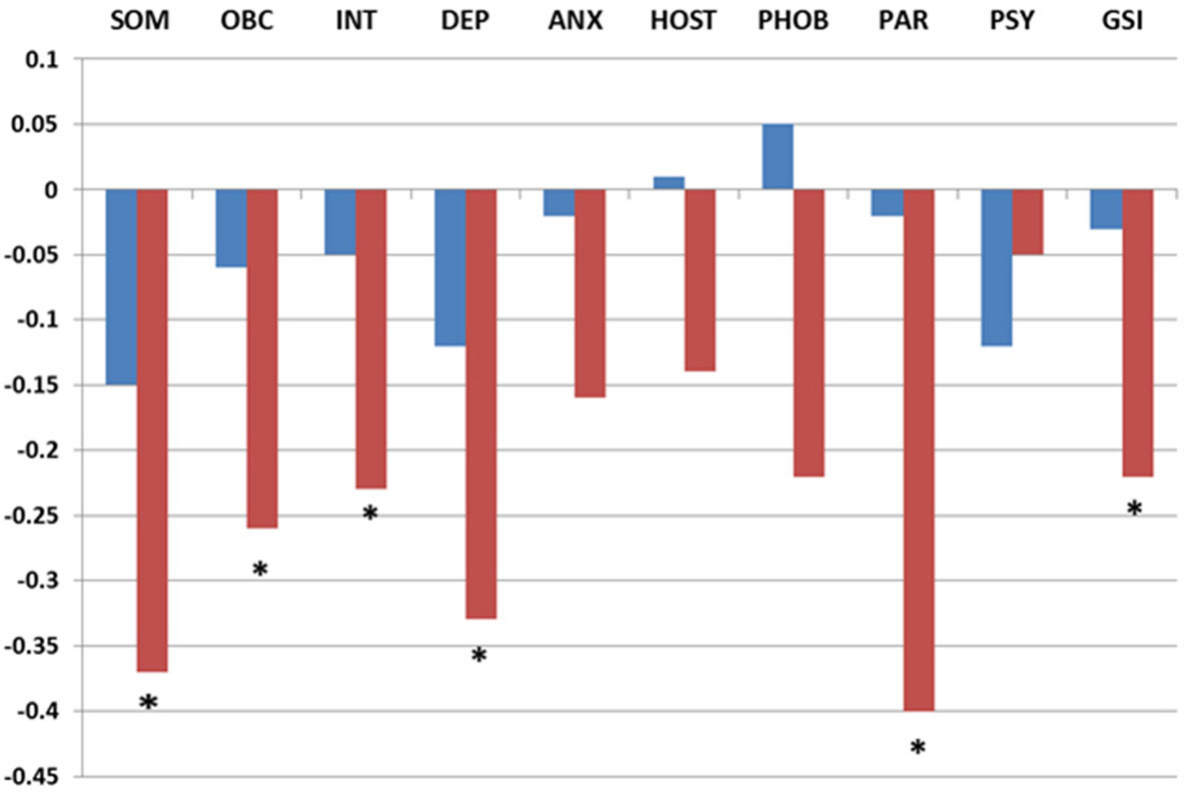
Changes in mental distress domains^a^ from discharge to the 6-month follow-up in patients treated for SUDs^b^. Notes: Red: the voluntary admission group; blue: the compulsory admission group. *p < 0.05 (paired *t*-test). ^a^Mental domains are subscores that correspond to the nine dimensions of the Symptoms Checklist (SCL-90-R). ^b^ Changes in mental distress were computed by subtracting the GSI determined at follow-up from the GSI determined at discharge; a ‘positive score change’ refers to an improvement in mental distress Abbreviations: SUD = substance use disorder, SOM = somatization, OBS = obsessive-compulsion, INT = interpersonal sensitivity, DEPR = depression, ANX = anxiety, HOST = hostility, PHOB = phobic anxiety, PARA = paranoid ideation, PSY = psychoticism, GSI = global score index

At the 6-month follow-up, the mental distress in the CA group had deteriorated to a level similar to that observed before treatment. In contrast, the VA group retained the improvement achieved during treatment throughout the follow-up (Fig. 1). The negative development in mental distress in the CA group seemed to arise mainly from increases in the following subscales: somatization (MD = 0.37, t(34) = -3.17, *p* = 0.003), obsessive-compulsive symptoms (MD = 0.26, t(34) 0 -2.91, *p* = 0.006), interpersonal sensitivity (MD = 0.23, t (34) 0 -2.16, *p* = 0.038), depression (MD = 0.33, t(34) = -3.13, *p* = 0.004), and paranoia (MD = 0.4, t(34) = -3.01, *p* = 0.005) (Fig. 2). For the sample as a whole, there was an overall decrease in mental distress from admission to follow-up (MD = 0.26, t(96) = 3.45, *p* < 0.001, CI: 0.112-0.416). The main contribution came from the progress achieved in the VA group (Fig. 1).

Preliminary bivariate regression analysis showed that there were no strong association (p-value >0.2) between partnerships, education, injection use the last 6 months, days in controlled environment, and changes in GSI from baseline to follow-up. These variables were not retained in multiple regression analysis. We found that only abstinence was a significant predictor for changes in GSI in the final adjusted model (β = 0.26, 95% CI 0.06–0.51; Table 2). Descriptively, 41% of the sample (40 of 97 patients) reported abstinence at follow-up; those that reported non-abstinence had only a minimal GSI improvement (0.04); in contrast, those that reported abstinence had a considerable GSI improvement of 0.58.

**Table 2:** Predictors of changes in mental distress in patients with SUDs at follow-up (*N* = 97)^a^

Predictor	Beta (95% CI)^b^	*P*-value
Socio-demographic variables		
Gender (female)	0.10 (-0.22/0.340)	0.561
Age, years	-0.01 (-0.04/0.01)	0.248
Compulsory admission	-0.28 (-0.60/0.05)	0.100
Duration of most problematic substance use, years	0.02 (-0.01/0.05)	0.110
Follow-up variables		
Abstinence in the last 30 days of follow-up	0.49 (0.18/0.80)	0.002

^a^ Mental distress was measured with the Global Score Index (GSI) of the Symptoms Checklist (SCL-90-R). Changes in mental distress were computed by subtracting the GSI determined at follow-up from the GSI determined at admission; a ‘positive score change’ refers to an improvement in mental distress

^b^ Unstandardized beta coefficient with 95% Confidence Interval (CI) and R^2^ = 0.1, derived from a multiple linear regression with simultaneous entry of variables (the "enter" method)

## Discussion

In the present study, the majority of in-patients with SUDs had mental distress levels above the clinical cut-off at baseline, but they improved during treatment. At the 6-month follow-up, the level of mental distress in the CA group returned to the level observed prior to treatment, but the VA group retained the improvement achieved with treatment. A multiple linear analysis identified active drug use as the only variable that could predict increased levels of mental distress at follow-up.

At baseline, the levels of mental distress were higher among patients in the VA group than among those in the CA group. However, the markedly elevated mental distress levels we observed among all patients with SUDs at admission confirmed findings from previous studies that showed that patients with SUDs experienced high level of mental distress compared to the general population [11]. This observation was also reported in a study that included patients legally coerced into treatment; moreover, patients in the coerced group had higher substance use severity and less mental distress than patients in the VA group, similar to our findings [34]. The high level of mental distress among patients in the VA group might have been an important motivating factor for voluntarily seeking treatment, as suggested in previous studies [49, 50]. Our findings indicated that compulsory treatment was primarily applied due to the severity of substance use, and not because of the severity of psychiatric symptoms. This finding appeared to be consistent with the NMHCA, a special law that governs treatment for patients with severe or life-threatening SUD conditions.

Our study showed that, after discharge, patients in the CA and VA groups followed divergent trajectories of mental distress. The VA group maintained reduced mental distress at the 6-month follow-up; this outcome was more positive than the outcomes previously reported in comparable studies [51]. In contrast, the CA group showed increases in depression, obsessive-compulsive symptoms, paranoia, somatization, and interpersonal sensitivity. This outcome appeared to have resulted from a relapse to drug use on a group level. This could suggest that those who sought treatment voluntarily may have been motivated and ready to make changes in substance use whereas those who were compulsorily admitted may not to the same extent have seen their drug use as a problem or were not ready to consider reducing use. This might highlight the need for extra supports to build motivation toward change within the CA group (both during the inpatient stay and as part of aftercare).

Patients that used injected drugs exhibited higher rates of mental distress, consistent with previous findings [52]. This finding suggested that the severity of a SUD was related to the level of mental distress. A previous paper reported improved 6-month follow-up SUD outcomes of the present study (frequency of substance use, injection use, and overdoses), but improved outcomes were reported significantly more frequently in the VA than in the CA group (e.g., 61% versus 37% reported reduction in the frequency of the preferred substance) [37]. The present paper elaborate on these findings; patients that remained abstinent were more likely to show lower levels of mental distress than patients that relapsed at 6 month follow-up.. In accordance with other studies [53, 54], our findings implied that patients that actively used drugs were less likely to retain the improvement in mental distress achieved with treatment; this finding highlighted the complex nature of mental distress in patients with SUDs. Epidemiological studies on the general population have shown that there are bi-directional influences between SUDs and psychiatric comorbidity; these two conditions negatively influence each other [15].

In light of the limited formalized care after discharge, it is possible that the inpatient-period alone was insufficient to establish long-term abstinence. Considering that the models for case management share the same core elements: assessment, planning, linkage and monitoring [55, 56], it may be worth noticing that an alternative may include specifically designed approaches for CA patients based on the assumption that they may be ambivalent about change such as for example enhanced use of motivational interview in the monitoring phase.

A continuum of care that included after-discharge care was previously demonstrated to be supportive in retaining improvements up to follow-up among patients that underwent CA [56].

Our findings suggested that both providers and programs should be available to provide assessments and management of co-existing psychological problems. This initiative should be built into the care period after discharge. Stand-alone interventions should not be considered adequate treatment for individuals with severe SUDs [57].

In the acute phase of CA treatment, the main goal is to provide life-saving treatment; however, in the longer term, the aim is to reduce drug use and increase the motivation for further treatment, preferably voluntary treatment, which can lead to long-term recovery [58]. The NMHCA does seem to fulfil its aim of reaching patients with severe SUDs. To obtain maximum long-term benefits for patients that undergo CA, the in-patient treatment should be more integrated into the broader treatment system. Accordingly, from our standpoint, a key factor for achieving maximum benefit is to achieve better coordination between the various care services; and in our case, this factor is particularly important during the transition to care after discharge. For example, this can be achieved by a case management-based approach [59].

### Methodological considerations

The strength of this study was its prospective design, which allowed the examination of psychiatric distress over time; i.e., the 6 months following discharge from SUD treatment. However, caution should be taken in generalizing these findings, because there was a high-attrition rate at follow-up. In addition, this study was based on self-reported data. Although the dataset is largely representative of hospitalized SUD populations in Norway, some data, particularly the outcomes in the CA group, may vary considerably across settings and regions with different laws regarding compulsory SUD treatment outside of Norway. Although longitudinal studies like this can enhance causal inference it cannot eliminate competing explanations and, as a result, does not establish a causal relationship.

## Conclusions

This study profiled the mental distress in two types of patients treated for SUDs. One type of patient was admitted voluntarily. These patients had high levels of mental distress at baseline, which improved during treatment; moreover, this improvement was maintained at the 6-month follow-up. The other type of patient underwent compulsory treatment. These patients had lower mental distress than the patients voluntarily admitted, but they also showed improvements with treatment; however, in the CA group, the levels of mental distress had returned to baseline at the 6-month follow-up.

Our study found that active substance use was the sole predictive factor of negative change in mental distress in patients with SUDs at the 6-month follow-up visit. This finding highlighted the importance of abstinence as a treatment goal for individuals with severe SUDs, also in order to maintain mental health stability.

This study also highlighted the need to employ a broader range of after-discharge interventions to prevent relapses and accompanying increases in mental distress for patients that undergo CA. In addition to the formalized treatment options available, clinicians may recommend that patients seek abstinence-supportive help, for example, from peer-based groups [60], to maintain improvements in mental distress achieved during treatment.

## Abbreviations

CA: Compulsory admitted
CI: Confidence intervals
EuropASI: European addiction severity index
ICD-10: International classification of diseases and related health problems
NMHCA: Norwegian municipal health care act
OR: Odd ratio
SUD: Substance use disorders
VA: Voluntarily admitted

## References

[1] Stinson FS, Grant BF, Dawson DA, Ruan WJ, Huang B, Saha T (2005). Comorbidity between DSM-IV alcohol and specific drug use disorders in the United States: Results from the National Epidemiologic Survey on Alcohol and Related Conditions. Drug Alcohol Depend.

[2] Flynn PM, Brown BS (2008). Co-occurring disorders in substance abuse treatment: Issues and prospects. J Subst Abuse Treat.

[3] Hall W, Teesson M, Lynskey M, Degenhardt L (1999). The 12-month prevalence of substance use and ICD-10 substance use disorders in Australian adults: Findings from the National Survey of Mental Health and Well-Being. Addiction.

[4] Hunt GE, Bergen J, Bashir M (2002). Medication compliance and comorbid substance abuse in schizophrenia: Impact on community survival 4 years after a relapse. Schizophr Res.

[5] Scott H, Johnson S, Menezes P, Thornicroft G, Marshall J, Bindman J, Bebbington P, Kuipers E (1998). Substance misuse and risk of aggression and offending among the severely mentally ill. Br J Psychiatry.

[6] Appleby L, Shaw J, Amos T, McDonnell R, Harris C, McCann K, Kiernan K, Davies S, Bickley H, Parsons R (1999). Suicide within 12 months of contact with mental health services: National clinical survey. Br Med J.

[7] Schafer I, Eiroa-Orosa FJ, Verthein U, Dilg C, Haasen C, Reimer J (2010). Effects of psychiatric comorbidity on treatment outcome in patients undergoing diamorphine or methadone maintenance treatment. Psychopathology.

[8] Opsal A, Clausen T, Kristensen O, Elvik I, Joa I, Larsen TK (2011). Involuntary hospitalization of first-episode psychosis with substance abuse during a 2-year follow-up. Acta Psychiatr Scand.

[9] Mills KL, Deady M, Teesson M, Sannibale C, Proudfoot H, Burns L, Mattick R (2012). Guidelines on the management of co-occurring mental health conditions in alcohol and other drug treatment settings: How useful are they?. Mental Health Substance Use: Dual Diagnosis.

[10] Dickey B, Azeni H (1996). Persons with dual diagnoses of substance abuse and major mental illness: Their excess costs of psychiatric care. Am J Public Health.

[11] Landheim AS, Bakken K, Vaglum P (2006). Impact of comorbid psychiatric disorders on the outcome of substance abusers: A six year prospective follow-up in two Norwegian counties. BMC Psychiatry.

[12] Olsen LR, Mortensen EL, Bech P (2006). Mental distress in the Danish general population. Acta Psychiatr Scand.

[13] Olsson TM, Fridell M (2015). Women with comorbid substance dependence and psychiatric disorders in Sweden: a longitudinal study of hospital care utilization and costs. BMC Health Serv Res.

[14] Kessler RC, Chiu WT, Demler O, Walters EE (2005). Prevalence, severity, and comorbidity of 12-month DSM-IV disorders in the National Comorbidity Survey Replication. Arch Gen Psychiatry.

[15] Alonso J, Angermeyer MC, Bernert S, Bruffaerts R, Brugha TS, Bryson H, de Girolamo G, Graaf R, Demyttenaere K, Gasquet I (2004). Prevalence of mental disorders in Europe: results from the European Study of the Epidemiology of Mental Disorders (ESEMeD) project. Acta Psychiatr Scand Suppl.

[16] Jane-Llopis E, Jané-Llopis E, Matytsina I, Jané-Llopis E, Matytsina I (2006). Mental health and alcohol, drugs and tobacco: a review of the comorbidity between mental disorders and the use of alcohol, tobacco and illicit drugs. Drug and alcohol review.

[17] Kessler RC, Nelson CB, McGonagle KA, Edlund MJ, Frank RG, Leaf PJ (1996). The epidemiology of co-occurring addictive and mental disorders: implications for prevention and service utilization. Am J Orthopsychiatry.

[18] Hoxmark E, Benum V, Friborg O, Wynn R (2010). Reduction in mental distress among substance users receiving inpatient treatment. Intl Journal of Mental Health Syst.

[19] Wynn R (2007). Prior psychotic episodes among patients in a substance abuse clinic. J Substance Use.

[20] Hattenschwiler J, Ruesch P, Modestin J (2001). Comparison of four groups of substance-abusing in-patients with different psychiatric comorbidity. Acta Psychiatr Scand.

[21] Pasareanu AR, Opsal A, Vederhus JK, Kristensen O, Clausen T (2015). Quality of life improved following in-patient substance use disorder treatment. Health Quality Life Outcomes.

[22] Kaskutas LA, Borkman TJ, Laudet A, Ritter LA, Witbrodt J, Subbaraman MS, Stunz A, Bond J (2014). Elements that define recovery: the experiential perspective. Journal of Studies Alcohol Drugs.

[23] McLellan A, McKay JR, Forman R, Cacciola J, Kemp J (2005). Reconsidering the evaluation of addiction treatment: From retrospective follow-up to concurrent recovery monitoring. Addiction.

[24] Kleber HD, Weiss R, Anton R, George T, Greenfield S, Kosten T, O'Brien C, Rounsaville B, Strain E, Ziedonis D (2007). Treatment of patients with substance use disorders. Am J Psychiatr.

[25] Ministry of Health and Care Services (MHCS). The Norwegian Public Health Act. In*.* Edited by MHCS; 2011.

[26] Urbanoski KA (2010). Coerced addiction treatment: Client perspectives and the implications of their neglect. Harm Reduction J.

[27] Broadstock M, Brinson D, Weston A, Collaboration HSA. The effectiveness of compulsory, residential treatment of chronic alcohol or drug addiction in non-offenders: a systematic review of the literature: Health Services Assessment Collaboration (HSAC), University of Canterbury; 2008.

[28] Wild TC (2006). Social control and coercion in addiction treatment: towards evidence‐based policy and practice. Addiction.

[29] Stevens A, Berto D, Heckmann W, Kerschl V, Oeuvray K, van Ooyen M, Steffan E, Uchtenhagen A (2005). Quasi-compulsory treatment of drug dependent offenders: An international literature review. Subst Use Misuse.

[30] Brecht ML, Greenwell L, Von Mayrhauser C, Anglin MD (2006). Two-year outcomes of treatment for methamphetamine use. J Psychoactive Drugs.

[31] Gerdner A, Berglund M. Compulsory care of subtance misuse-effect and quality; in Goverments Task Force on Substance Misuse, Knowledge, Care. In*.* Edited by Affairs MoHaS. Stocklom; 2011: 653–770

[32] Rawson RA, Huber A, McCann M, Shoptaw S, Farabee D, Reiber C, Ling W (2002). A comparison of contingency management and cognitive-behavioral approaches during methadone maintenance treatment for cocaine dependence. Arch Gen Psychiatry.

[33] Wickizer TM, Campbell K, Krupski A, Stark K (2000). Employment outcomes among AFDC recipients treated for substance abuse in Washington State. Milbank Q.

[34] Copeland J, Maxwell JC (2007). Cannabis treatment outcomes among legally coerced and non-coerced adults. BMC Public Health.

[35] Kline A (1997). Profiles of criminal-justice clients in drug treatment: implications for intervention. Addict Behav.

[36] Kelly JF, Finney JW, Moos R (2005). Substance use disorder patients who are mandated to treatment: characteristics, treatment process, and 1- and 5-year outcomes. J Subst Abuse Treat.

[37] Pasareanu AR, Vederhus J-K, Opsal A, Kristensen Ø, Clausen T (2016). Improved drug-use patterns at 6 months post-discharge from inpatient substance use disorder treatment: results from compulsorily and voluntarily admitted patients. BMC Health Serv Res.

[38] Sheehan DV, Lecrubier Y, Sheehan KH, Amorim P, Janavs J, Weiller E (1998). The Mini-International Neuropsychiatric Interview (M.I.N.I.): the development and validation of a structured diagnostic psychiatric interview for DSM-IV and ICD-10. J Clin Psychiatry.

[39] Kokkevi A, Hartgers C (1995). EuropASI: European adaptation of a multidimensional assessment instrument for drug and alcohol dependence. Eur Addict Res.

[40] McLellan AT, Kushner H, Metzger D, Peters R, Smith I, Grissom G, Pettinati H, Argeriou M (1992). The Fifth Edition of the Addiction Severity Index. J Subst Abuse Treat.

[41] Derogatis LR, Lipman RS (1973). SCL-90: An outpatient psychiatric rating scale, preliminary report. Psychol Bull.

[42] Haver B (1986). The DSM-III diagnosis of alcohol use disorders in women: Findings from a follow-up study of 44 female alcoholics. Acta Psychiatr Scand.

[43] Barth K, Nielsen G, Havik OE, Haver B, Molstad E, Rogge H, Skatun M, Noklebye Heiberg A, Ursin H (1988). Assessment for three different forms of short-term dynamic psychotherapy. Findings from the Bergen Project. Psychother Psychosom.

[44] Mehlum L, Friis S, Irion T, Johns S, Karterud S, Vaglum P, Vaglum S (1991). Personality disorders 2-5 years after treatment: A prospective follow-up study. Acta Psychiatr Scand.

[45] Vassend O, Lian L, Andersen HT. Norske versjoner av NEO-Personality Inventory, Symptom Checklist 90 Revised og Giessen Subjective Complaints List. Del I. Tidsskrift for Norsk Psykologforening. 1992;29(12):1150–60.

[46] Fridell M, Hesse M (2006). Psychiatric severity and mortality in substance abusers: A 15-year follow-up of drug users. Addict Behav.

[47] Derogatis LR. Administration, scoring & procedures manual-II for the R (revised) version and other instruments of the psychopathology rating scale series. In: Clinic Psychometric Research. edn. Towson; 1983.

[48] Altman DG (1991). Practical statistics for medical research.

[49] Mojtabai R (2005). Use of specialty substance abuse and mental health services in adults with substance use disorders in the community. Drug Alcohol Depend.

[50] Marlowe DB, Kirby KC, Merikle EP, Festinger DS, McLellan AT (2001). Multidimensional assessment of perceived treatment-entry pressures among substance abusers. Psychol Addict Behav.

[51] Bakken K, Landheim AS, Vaglum P (2007). Axis I and II disorders as long-term predictors of mental distress: A six-year prospective follow-up of substance-dependent patients. BMC Psychiatry.

[52] Kidorf M, King VL, Peirce J, Burke C, Kolodner K, Brooner RK (2010). Psychiatric distress, risk behavior, and treatment enrollment among syringe exchange participants. Addict Behav.

[53] Ritsher JB, McKellar JD, Finney JW, Otilingam PG, Moos RH (2002). Psychiatric comorbidity, continuing care and mutual help as predictors of five-year remission from substance use disorders. J Stud Alcohol.

[54] Tomasson K, Vaglum P (1998). Psychiatric co-morbidity and aftercare among alcoholics: a prospective study of a nationwide representative sample. Addiction.

[55] Siegal HA, Rapp RC, Li L, Saha P, Kirk KD (1997). The role of case management in retaining clients in substance abuse treatment: An exploratory analysis. J Drug Issues.

[56] Lindahl ML, Berglund M, Tonnesen H (2013). Case management in aftercare of involuntarily committed patients with substance abuse. A randomized trial. Nordic J Psychiatry.

[57] Clausen T (2015). Coherent long-term treatment approaches–superior in the treatment of opioid dependence. Addiction.

[58] Lundeberg IR, Mjåland K, Søvig KH. Tvang i rusfeltet - Regelverk, praksis og erfaringer med tvang: Gyldendal Juridisk; 2014.

[59] Hesse M, Vanderplasschen W, Rapp R, Broekaert E, Fridell M. Case management for persons with substance use disorders. Cochrane Library. 2007;4: Art. No.: CD006265. doi: 10.1002/14651858.CD006265.pub2. 10.1002/14651858.CD006265.pub2 17943902

[60] Vederhus JK, Timko C, Kristensen O, Hjemdahl B, Clausen T (2014). Motivational intervention to enhance post-detoxification 12-Step group affiliation: a randomized controlled trial. Addiction.

